# Genetic tango: unveiling maize’s endosperm filling regulation orchestrated by NAKED ENDOSPERM1, NAKED ENDOSPERM2, and OPAQUE2

**DOI:** 10.1093/plcell/koad245

**Published:** 2023-09-27

**Authors:** Maryam Rahmati Ishka

**Affiliations:** Assistant Features Editor, The Plant Cell, American Society of Plant Biologists; Boyce Thompson Institute, Ithaca, NY, USA

The cereal endosperm is a vital energy store for germination and growth and serves as a key resource for human and animal consumption and industry. After fertilization, the triploid endosperm experiences coenocytic growth (∼1 to 3 days after pollination [DAP]) and cellularization (∼3 to 4 DAP), followed by differentiation (∼4 DAP). This phase gives rise to diverse cell types with unique features and functions. Roughly spanning 8 to 16 DAP, a pivotal shift occurs, transitioning from cellular growth to grain filling. In orchestrating this shift, transcription factors (TFs) NAKED ENDOSPERM1 (NKD1), NAKED ENDOSPERM2 (NKD2), and OPAQUE2 (O2) are key players (Schmidt et al. 1990). In this issue of *The Plant Cell*, **Hao Wu and colleagues** (Wu et al. 2023) found the intricate regulatory connections among these 3 TFs, as these factors collaboratively control gene networks during the shift from cellular development to grain-filling in maize endosperm (Fig.).

**Figure.**
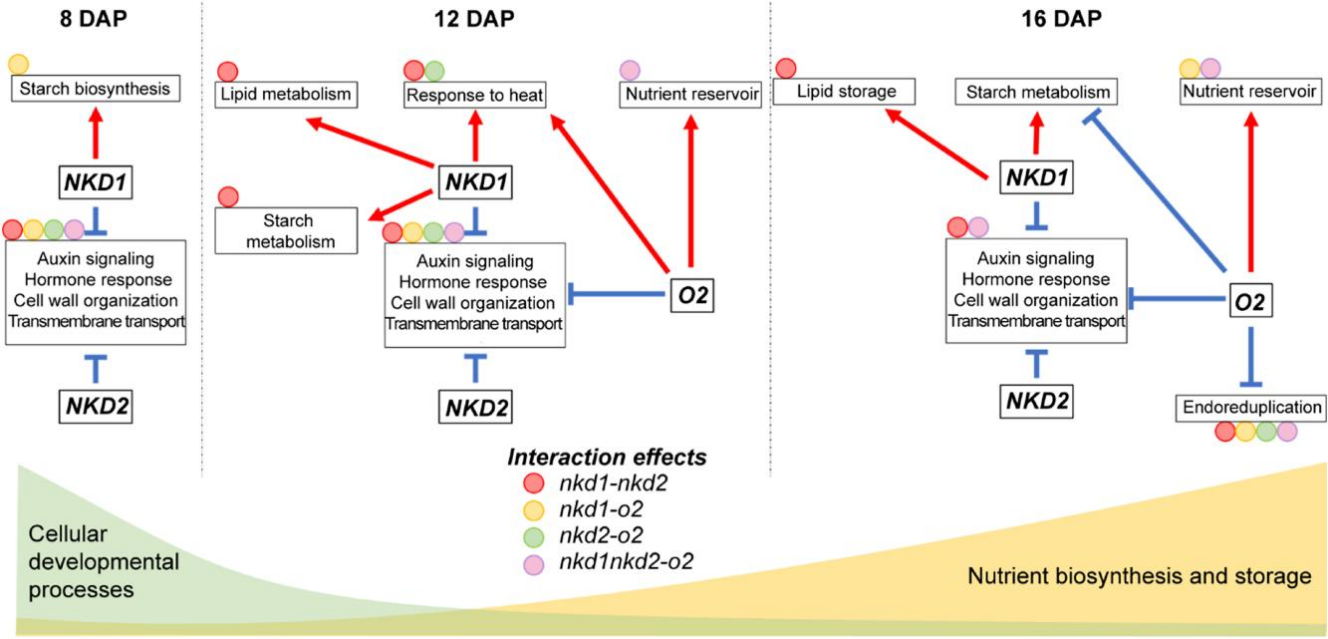
Model describing the regulatory function of NKD1, NKD2, and O2 on the transitions occurring during endosperm development. The processes are depicted within boxes, with red arrows indicating positive regulation, blue T-shaped lines indicating negative regulation, and interactions denoted by color-coded circles connected to their respective boxes. Adapted from Wu et al. (2023), Figure 8.

Previous studies have shown that NKD1, NKD2, and O2 are involved in grain-filling and exhibit interconnected relationships. *nkd1* and *nkd2* double mutants (*nkd1;2*) impact endosperm development and cause multiple compromised peripheral cell layers. This suggests that NKD1 and NKD2 restrict cell count while promoting differentiation. Also, in grain-filling, NKD1 and NKD2 positively influence storage protein genes. The *nkd1;2* mutant displays reduced starch, changes in starch branching, and irregular granules (Gontarek et al. 2016). Mutations in *o2* lead to an opaque kernel appearance by reducing storage protein levels. Target genes of *O2* are primarily related to nutrient reservoir functions like zein storage proteins, establishing its significance during grain filling. Single *nkd1* or *nkd2* mutants show increased expression of *NKD2* or *NKD1*, respectively (Yi et al. 2015). NKD2 directly represses *NKD1* expression, forming a feedback loop that could regulate NKD TF levels. *NKD1* and *NKD2* activate *O2* while *nkd1;2* mutants downregulate *O2* (Gontarek et al. 2016). *O2* activates *NKD2* and, together with *NKD2*, co-activates genes important for storage protein biosynthesis.

Wu et al. (2023) generated homozygous mutant combinations for *nkd1*, *nkd2*, and *o2* in a W22 inbred background to investigate their combined impact. The *o2* mutants displayed kernels of normal size but reduced translucency. The *nkd1;2* double mutant exhibited small, wrinkled kernels with reduced translucency. Remarkably, the *nkd1;nkd2;o2* triple mutant showed a highly shrunken, wrinkled phenotype. Furthermore, the *nkd1;o2* double-mutant kernels displayed a unique indentation below the crown region, which was absent in other genotypes. These observations imply an interaction between the *NKD1*, *NKD2*, and *O2* genes during grain development.

RNAseq was performed on developing endosperm tissues from each genotype from 8 DAP to 16 DAP to compare gene regulatory networks among the 3 TFs. The analysis showed that the 8-DAP transcriptomes were distinctly separated from later samples, indicating a significant shift between 8 and 12 DAP as the endosperm progresses from initial cell processes to grain-filling. Genotype impact was more pronounced at 12 and 16 DAP than at 8 DAP. Notably, genotypes with *o2* gene mutations were clearly separated from *O2+* genotypes (homozygous wild-type *O2* allele) at 12 and 16 DAP, highlighting the vital role of *O2+* in grain-filling. As expected, double-mutant *nkd1;2* genotypes consistently differed from single mutants and *Nkd1+;Nkd2+* genotypes due to redundancy. Triple-mutant *nkd1;nkd2;o2* transcriptomes clustered with earlier time points, possibly indicating delayed development in the triple-mutant endosperm. The authors then deployed Weighted Gene Co-expression Network Analysis to explore the relationships between the 3 TFs. From this analysis, the differentially expressed genes were sorted into co-expression modules, linked to *nkd1*, *nkd2*, and/or *o2* expression. At 8 DAP, *nkd1* exhibited a positive correlation with module that linked to starch metabolism. Conversely, at 16 DAP, *nkd2* and *o2* collaboratively influenced the module that is associated with storage protein production.

DNA Affinity Purification Sequencing (DAP-seq) was conducted on in vitro–produced NKD1 and NKD2 proteins to identify the DNA regulatory elements and potential target genes of these 2 TFs. This analysis showed that there was a significant overlap between DAP-seq targets of NKD2 and differentially expressed genes associated with *nkd1*, *o2*, *nkd1;o2*, and *nkd1;nkd2;o2*. Additionally, hub genes were identified, indicating strong connectivity within O2, NKD1, and NKD2. O2’s targeted hub genes encompassed TFs like *bZip17* and *GBF1*, underlining O2’s hierarchical role. Among possible *NKD2* targets were *ARF1* and *ARF29*.

Together, Wu et al. (2023) demonstrate NKD1, NKD2, and O2 function dynamically, regulating functions primarily associated with cellular development at early stages and transitioning to functions associated with nutrient accumulation and storage during grain-filling in maize. These functions have been demonstrated at both the phenotypic and transcriptomic levels. They identified gene co-expression modules that are associated with biological processes, along with hub genes including potential direct targets of NKD1, NKD2, and O2.

## Dive Curated Terms

The following phenotypic, genotypic, and functional terms are of significance to the work described in this paper:

GBF1 Gramene: AT4G36730
GBF1 Araport: AT4G36730

BZIP17 Gramene: AT2G40950
BZIP17 Araport: AT2G40950

dna Gramene: EO:0007532
dna Araport: EO:0007532

ARF1 Gramene: AT1G59750
ARF1 Araport: AT1G59750
